# *QuickStats:* Age-Adjusted Percentages* of Adults Aged 18–64 Years Who Never Felt Rested in the Past Week,^†^ by Sex, Race, and Hispanic Origin^§^ — National Health Interview Survey,^¶^ 2017–2018

**DOI:** 10.15585/mmwr.mm6842a4

**Published:** 2019-10-25

**Figure:**
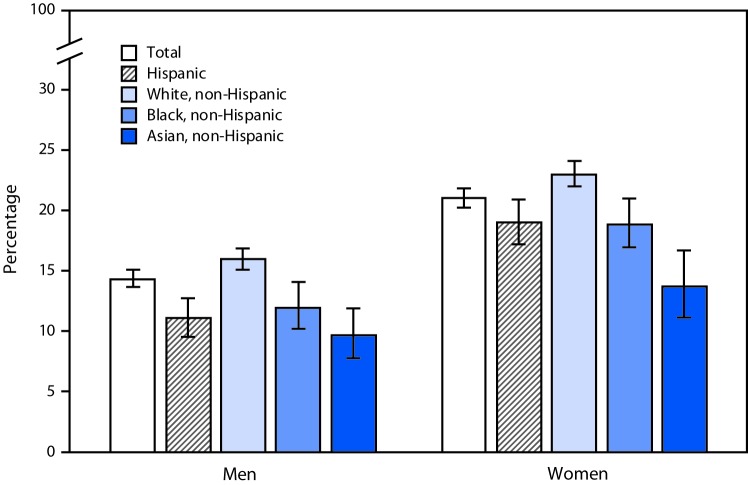
During 2017–2018, among persons aged 18–64 years, women were more likely than men to report they never felt rested in the past week overall (21.1% versus 14.3%) and in each race and Hispanic origin group. Non-Hispanic white men (16.0%) were more likely to report they never felt rested than were Hispanic men (11.1%), non-Hispanic black men (12.0%), and non-Hispanic Asian men (9.7%). Non-Hispanic white women (23.0%) were more likely to report they never felt rested than were Hispanic women (19.0%), non-Hispanic black women (18.9%), and non-Hispanic Asian women (13.7%).

**Source:** National Center for Health Statistics, National Health Interview Survey, 2017–2018. https://www.cdc.gov/nchs/nhis.htm.

